# Carbon Paste Electrode Modified With Cuo–Nanoparticles as a Probe for Square Wave Voltammetric Determination of Atrazine

**DOI:** 10.17795/jjnpp-9985

**Published:** 2013-07-27

**Authors:** Nadereh Rahbar, Hooshang Parham

**Affiliations:** 1 Nanotechnology Research Center, School of Pharmacy, Ahvaz Jundishapur University of Medical Sciences, Ahvaz, IR Iran; 2 Department of Medicinal Chemistry, School of Pharmacy, Ahvaz Jundishapur University of Medical Sciences, Ahvaz, IR Iran; 3 Department of Chemistry, Shahid Chamran University, Ahvaz, IR Iran

**Keywords:** Atrazine, Nanoparticles, Cupric Oxide

## Abstract

**Background:**

Atrazine (ATZ) is a widely used herbicide in most countries because of its low cost and good selectivity. The concentration of ATZ that the EPA considers safe to consume in drinking water is 3 ppb. Therefore, recently, there have been concerns about its determination in trace levels. This compound is not electro-active, so in this research indirect electrochemical method for its detection in low levels was proposed.

**Objectives:**

The main aim of this study is the indirect determination of ATZ in water samples by voltammetry using nano-particle modified electrode.

**Materials and Methods:**

A nano-CuO modified carbon paste electrode (NMCPE) is constructed and its application for indirect square wave voltammetric (SWV) detection of ATZ is reported. The sensing performance mechanism of the nano-CuO modified carbon paste electrode toward atrazine is due to complexation of the analyte with Cu (II) ion. The peak current for copper (II) reduction decreases with increase in the ATZ concentration and is monitored for its determination. Instrumental and chemical parameters influencing the detection of ATZ were optimized.

**Results:**

The results revealed that decrease in peak current was proportional to ATZ concentration over the range of 5-75 ng/mL. The limit of detection (LOD) and limit of quantification (LOQ) were 2 ng/mL and 5.6 ng/mL (n = 20), respectively. The relative standard deviation (n = 10) for the determination of 10 and 50 ng/mL of ATZ solution was estimated as 4.9% and 4.2 %, respectively.

**Conclusions:**

This easily fabricated electrode together with the fast and sensitive SW voltammetry was successfully applied for the determination of concentration of ATZ at trace levels, in different water samples.

## 1. Background

Atrazine (2-chloro-4-ethylamino 6-terbutylamino 1, 3, 5 triazine) herbicide is widely used in most countries for selective weed control in corn, wheat, barley, grain sorghum and sugar cane, but it is also widely employed for non-agricultural usage (railways and roadside verges). In recent years, there have been concerns about the level of ATZ herbicide runoff entering surface waters. Unfortunately ATZ is also one of the most commonly detected pesticides in most countries in ground and surface water. This concerns scientists and healthcare experts because ATZ has been linked to several kinds of health problems in humans and amphibians. Some of these health problems are: cancer, birth defects, reproductive effects, neurotoxicity, kidney and liver damages, sensitizer and irritant, groundwater contamination. This toxic chemical can also accumulate in fish. As a result of these concerns, use of this chemical has been restricted in some parts of the U.S. and around the world (1). According to the environmental protection agency (EPA), the maximum contaminant level (MCL) of ATZ for public drinking water is about 3 ng/mL (1). Because of such environmental problems, ATZ and other related herbicides are in the list of chemical pollutants that need to be more heavily monitored due to their toxicity, persistence, accumulation in the environment and their effects on the nature health (2, 3). The most widely used methods for the determination of triazine herbicides are complicated traditional chromatographic techniques including gas chromatography (GC) (4-10) and high-performance liquid chromatography (HPLC) (11-17). Nowadays, different environmental immunoassay techniques are also reported for detection of trace amounts of triazine herbicides family (18-24). However, these methods suffer from expensive and extensive pretreatment steps. Electroanalytical methods are known to attend the demand for minimal sample treatment, low consumption of organic solvents and good sensitivity (25). The determination of ATZ and other triazines have been studied with mercury electrodes by different voltammetric methods (26-29). However, these methods are becoming increasingly undesirable because of toxicity of mercury and most of the studies have been focused on fabrication and modification of different working electrodes to improve their sensitivity and lower the detection limit. Specific enzymes, organic compounds with electrochemical activity and nano-materials are usually employed as the modifying materials (30). Regarding this, carbon pastes undoubtedly represent one of the most convenient materials for the preparation of the modified electrodes by mixing a modifier with the paste (31). In this paper an indirect, sensitive and fast SWV method for the determination of ATZ using a laboratory constructed nano-CuO modified carbon paste electrode is reported.

## 2. Objectivs

The main aim of this study is the indirect determination of ATZ in water samples by voltammetry using nano-particle modified electrode.

## 3. Materials and Methods

### 3.1. Reagents and Solutions

All chemicals were of analytical grades and obtained from Merck (Darmstadt, Hesse, Germany). ATZ (purity 97%) was purchased from Fluka (Milwaukee, USA). Graphite powder (size < 100 µm) was purchased from Fluka (Milwaukee, USA). Copper oxide (CuO, 40-80 nm) was purchased from Inframat Advanced Materials (Farmington, CT, USA). Double distilled water was used throughout. ATZ stock solution (100 µg/mL) was prepared by dissolving 0.0103 g of ATZ in 20 mL of methanol and diluting to 100 mL. Then ATZ solutions in the concentration range of 5-75 ng/mL were prepared by successive dilution of stock solution. Acetate and phosphate buffer solutions (0.1 M) were prepared from CH_3_COONa, KH_2_PO_4_ and K_2_HPO_4_. HCl and NaOH solutions were used for pH adjustment of buffer solutions. For preparing of Britton-Rabinson buffer (0.1 M), appropriate amounts of ortho-phosphoric acid, acetic acid and boric acid was mixed.

### 3.2. Apparatus

All voltammetric measurements were carried out on an EG & G 273A electrochemical device (Princeton Applied Research, PCR, USA). A three-electrode arrangement was used throughout. A nano-CuO modified carbon paste as working electrode and a platinum wire as auxiliary electrode together with an Ag/AgCl reference electrode, using 3 M KCl as electrolyte with a porous membrane were used.

### 3.3. Fabrication of the Nano-CuO Modified Carbon Paste Electrodes

Nano-CuO modified carbon paste electrode was prepared by mixing graphite powder, paraffin and CuO-nanoparticles in a way that the ratio of CuO:C:Paraffin in each electrode was 5:65:30 w/w. The carbon paste electrode was finally obtained by packing the paste into an insulin syringe and arranged with a copper wire serving as an external electric conductor. 

### 3.4. Indirect Detection of ATZ

The sensing performance mechanism of the nano-CuO modified carbon paste electrode toward ATZ (analyte) is due to complexation of the analyte with Cu (II) ion (32, 33). By applying a negative potential, the nano-CuO particles are reduced to Cu and then oxidation of Cu to copper (II) ions by applying a positive potential is occurred. The peak current for reduction of copper (II) decreases with increasing the concentration of ATZ and difference between peak current intensities of ATZ and the blank solutions ∆I, is monitored for its determination. The general procedure adopted for indirect detection of trace amounts of ATZ was as follows: appropriate amounts of standard ATZ solution, 0.5 mL of 1 mol/L of KNO_3_ ¬¬¬¬¬¬ solution and 1 mL of 0.1 M acetate buffer (pH = 5.5) were transferred into the electrochemical cell. NMCPE with reference and auxiliary electrodes were dipped into the test solution and SWV was performed in three steps, namely:

Step 1: The potential -1.25 V was applied to the working electrode for 10 s to reduce the CuO-nano particles at the surface of the carbon paste electrode to Cu.

Step 2: The potential +0.80 V was applied to the working electrode for 5 s to oxidize Cu particles at the surface of the paste electrode (produced in the first step) to Cu (II) ions and then solution was stirred for three min in open circuit in order to ATZ accumulation.

Step 3: Cu (II) SW voltammograms were recorded by scanning a negative-going potential over the potential range from +0.50 to -0.50 V, with a pulse height potential of 25 mV and a frequency of 60 Hz. The decrease in peak current of Cu (II) SW voltammogram was monitored for indirect detection of ATZ. It must be mentioned that by increasing the concentration of ATZ in the test solution, the peak current intensity of Cu (II) decreases due to complexation with ATZ. The difference between peak current intensities (∆I at -0.10 V) of the test and the blank solutions is linear with ATZ concentration. In order to regenerate new and fresh surface on NMCPE, the tip of the electrode was polished using weighing paper after each determination. In the case of water samples, 0.5 mL of 1 mol/L of KNO_3_¬¬¬¬¬¬ solution was added to 8 mL of water sample and the pH was fixed to 5.5 by adding 1 mL of 0.1 M acetate buffer and then was diluted to 10 mL. The solution mixture was transferred to electrochemical cell and the recommended procedure is followed as mentioned above.

### 3.5. Method Validation

In order to validate the proposed method, precision, accuracy and linearity of the method were tested according to the FDA guideline (34). The reproducibility and repeatability (the intra-day and inter-day precision and accuracy) of the method were obtained by analysis of four replicates of each low, mid and high concentrations for standard solutions of ATZ on 5 consecutive days.

## 4. Results

**Figure 1.**
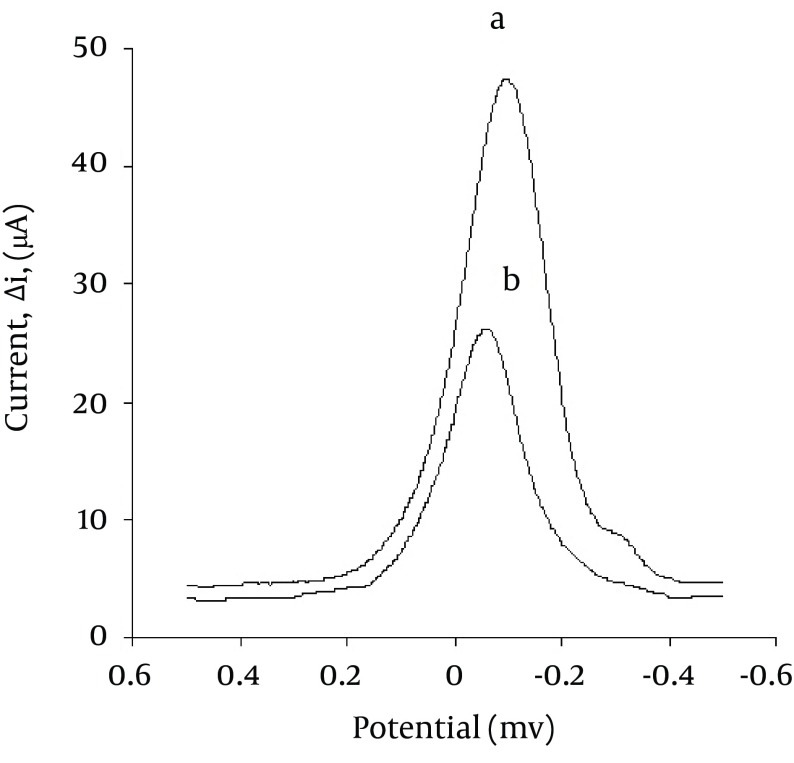
The SW Voltammograms of Cu (II) in the (a) Absence and (b) Presence of ATZ on Modified Carbon Paste Electrode Concentration of ATZ: 50.0 ng/mL; pH = 5; Accumulation Time: Three min; Conditioning Potential: -1.25 V; Conditioning Time: 10 sec; Pulse Height: 25 mV; Scan Increment: 2 mV; Frequency: 60 Hz.

The sensitive SWV method is chosen to determine trace quantities of ATZ in different water samples. Figure 1 shows sharp, symmetric and well-defined SW voltammograms of Cu (II) (at pH = 5.5) in the absence (a) and presence of ATZ (b). The peak current intensity of Cu (II) decreases with increasing ATZ concentration in the test solution. Different chemical and instrumental parameters such as electrode composition weight ratios, pH, accumulation time, accumulation potential, pulse height and frequency were optimized and a well shaped peak with maximum peak current for the determination of ATZ solutions was obtained. 

### 4.1. Electrode Composition

**Figure 2.**
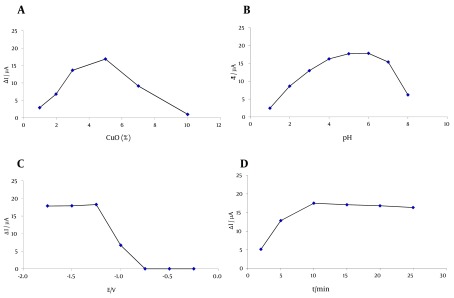
a) The Effect of the Amount of CuO-nanoparticles (w/w %) Added to Paste, b) The Effect of the Sample Solution PH, c) The Effect of Conditioning Potential and d) The Effect of Accumulation Time on the Peak Current Intensity Differences of Cu (II) Voltammograms in the Absence and Presence of ATZ (at -0.1 V) Conditions: Concentration of ATZ: 50 ng/mL, pulse height: 25 mV, frequency: 60 Hz.

The most sensitive and best weight ratio (w/w) of C: Paraffin oil for preparation of carbon paste electrode is 70:30 (w/w) (30). Mixtures with paraffin ratios more than 30% by weight show low conductivity and so no adequate voltammogram was obtained in such cases. In the cases where the paraffin ratio is less than 30%, the paste becomes dry (polishing process is hard and takes a long time) and no repeatable voltammogram was obtained. CuO-nanoparticles were used for modification of the paste. Different amounts of CuO-nanoparticles were added to the paste and mixed thoroughly on mortar for construction of the modified carbon paste electrode. Highest difference between peak current intensities of ATZ solution and the solution without ATZ (blank), ∆I, was achieved by CuO:C:Paraffin weight ratio of 5:65:30 and this weight composition was used for NMCPE construction in further works (Figure 2a). In cases of lower ratios than 5 for CuO, the intensity of peak current decreases due to lower amount of CuO and low accumulation of ATZ on the electrode surface. Higher amounts of CuO (> 5 g per 100 g of paste) cause decreasing of ∆I duo to too much increase of the blank peak current intensity. 

### 4.2. The Effect of pH

Britton-Robinson, acetate and phosphate buffer solutions were used to study the effect of pH of the test solution on the peak current intensity of Cu (II) voltammograms in the presence of a fixed concentration of ATZ (50 ng/mL). The results showed that by using acetate buffer in the pH range of 3.0 - 8.0, the highest ∆I was obtained at pH 5.5 (Figure 2b). The optimum volume of the buffer solution was 1 mL. At pH values lower than 5, protonation of ATZ molecules causes the increase of the peak current intensity of Cu (II) due to less complex formation between ATZ and Cu (II). 

### 4.3. The Type of Supporting Electrolyte

The effect of different salt solutions (as supporting electrolyte) on the peak current intensity of Cu (II) voltammograms in the presence of a fixed concentration of ATZ (50 ng/mL) was investigated. Sample solutions containing salts such as KCl, KNO_3_, NaCl and K_2_SO_4_ in the concentration range of 0.1-1 mol/L were tested. The results revealed that the highest ∆I was observed in the presence of 0.5 mL of 1.0 mol/L of KNO_3_ solution as supporting electrolyte.

### 4.4. Influence of Conditioning Potential and Time

In order to prepare the conditions for reaction of the analyte (ATZ) with Cu (II) at the electrode surface, a conditioning potential must be applied to the working electrode for reduction of CuO-nanoparticles to Cu (First step). This first conditioning potential must be applied to the electrode before applying the second conditioning potential for oxidation of Cu particles at the electrode surface to Cu (II), as mentioned in section 3.4 (Second step). Third step is scanning and recording the voltammograms of the remaining Cu (II) after formation of complex with ATZ. The effect of first conditioning potential and also conditioning time on ∆I, were investigated under the optimum chemical parameters. Different conditioning potentials (1st) were applied in the range between -0.25 V to -1.75 V. The results in Figure 2c show that at applied potentials smaller than -0.6 V, no considerable ∆I was observed. ∆I, increases with increasing the applied potential to -1.25 V and remains almost constant after this potential. The applied potential of -1.25 V was used throughout as the optimum conditioning potential for reduction of CuO-nanoparticles to Cu at the electrode surface. In order to obtain the optimum conditioning time, the conditioning potential (-1.25 V) was applied in different time periods between 2 - 25 S. ∆I increases with increasing the conditioning time up to 10 S and remains almost constant after this time. Different conditioning potentials (2nd) were applied in the range between 0 and +1 V. The results show that at applied potentials smaller than +0.5 V, no considerable ∆I was observed. ∆I increases with increasing the applied potential from +0.5 V to +0.8 V and remains almost constant after this potential. The applied potential of +0.8 V was used throughout as the optimum conditioning potential for oxidation of Cu particles to Cu (II) ions at the electrode surface. ∆I increases with increasing the conditioning time up to 5 sec and remains almost constant after this time. 

### 4.5. Influence of Accumulation Time and Potential

The effect of accumulation time and potential on ∆I, was investigated under the optimum chemical parameters. Figure 2d shows that ∆I increase with accumulation time increasing from 0 to 180 sec. But when accumulation time exceeds 3 min, ∆I keeps almost unchanged, meaning that accumulation equilibrium is achieved at the electrode-solution interface. The influence of accumulation potential was examined from +0.3 to -0.5 V. The results showed that ∆I is almost independent of accumulation potential. Thus, accumulation of ATZ is performed under open-circuit. 

### 4.6. The Effect of Frequency and Pulse Height on Peak Current Intensity Differences

The effect of frequency changes on the voltammogram’s peak current intensity differences (at -0.10 V) was studied in the frequency range of 20-200 Hz. The results showed that frequencies in the range of 40-80 Hz give the highest difference in peak current intensities and so, 60 Hz was chosen as optimum frequency value. The effect of pulse height variation on the difference in peak current intensities, (∆I), was studied in the potential range of 15-125 mV. The obtained results showed that increasing the pulse heights up to 25 mV will cause an increase in (∆I). Pulse heights more than 25 mV cause broadening of voltammogram and so decreasing (∆I). So the optimum pulse height value was selected to be 25 mV.

### 4.7. The Effect of Interferences

The effect of interfering substances on maximum peak current was studied prior to the application of the method on real samples. The SWV determination of ATZ in the presence of interfering substances was investigated. A change of ± 5% in ∆I was defined as the tolerance limit. The results revealed that most of the studied substances show no interfering effect in the determination of ATZ in water samples. The cations such as Hg^2+^, Pb^2+^, Cd^2+^, Cu^2+^ and Cr^3+^ do interfere when their concentration are more than 10 fold with respect to ATZ because the redox potentials of these ions are near together. Furthermore some compounds such as simazine with similar structures do interfere with the concentrations in the same levels.

### 4.8. Analytical Performance and Method Validation

**Figure 3.**
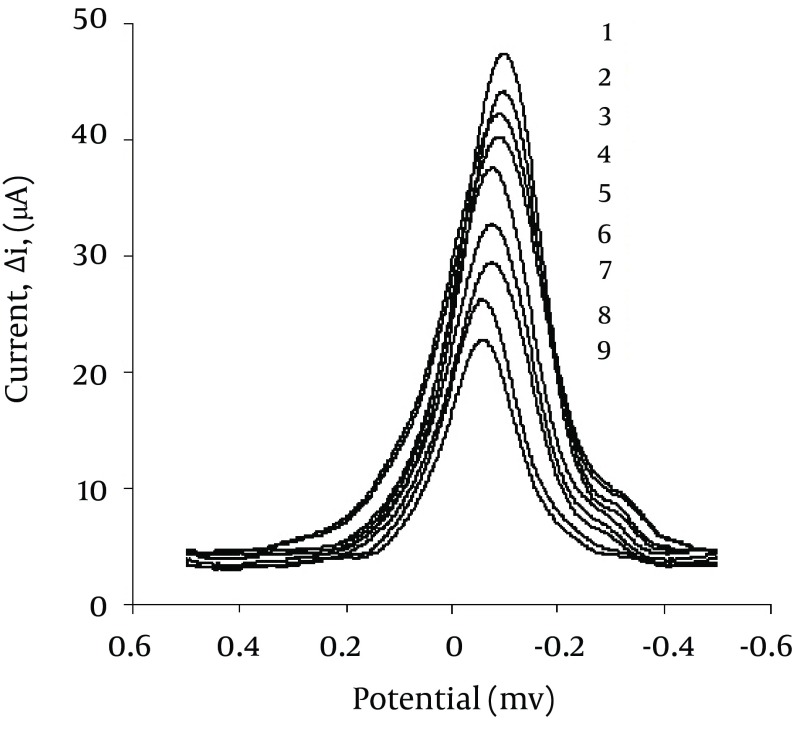
Square Wave Voltammograms of Cu (II), Under Optimum Conditions, in the Presence of Different Concentrations of ATZ in the Test Solution 1) 0; 2) 5; 3) 10; 4) 15; 5) 25; 6) 40; 7) 50; 8) 60; 9) 75 ng/mL

Analytical performance and validation of the proposed method was showed by studying the dynamic range, LOD, LOQ, accuracy and precision. Under optimal conditions, the calibration graph for the determination of ATZ is obtained in the concentration range of 5.0–75.0 ng/mL with a correlation coefficient of 0.9980. The regression equation for the line was ∆I = 0.3120 CATZ + 2.0938 (n = 9), where CATZ is the concentration of ATZ in ng/mL and (∆I) is the peak current intensity difference between the analytic solution and the blank voltammograms (Figure 3). The slope of the line b = 0.3120 ± 0.0176 which shows a RSD (relative standard deviation) = 5.3% and the intercept of the line a = 2.0938 ± 0.04263 with a RSD = 2.3% were also obtained. Under optimum experimental conditions, the limit of detection (LOD) and limit of quantification (LOQ), of the proposed method based on three and ten times the standard deviation of the blank (3Sb, 10Sb), were 2.0 ng/mL and 5.6 ng/mL (n = 9), respectively. 

Quality controlled (QC) samples of ATZ were used to show reproducibility and repeatability terms of the proposed method. Intra and inter-day precision and accuracy data for SW voltammetric detection of ATZ in spiked standard solutions are summarized in Table 1. As results show RSD% values for inter-day and intra-day runs are reasonable. The recovery values from spiked standard solutions containing low, medium and high concentrations of ATZ (10, 30 and 60 ng/mL) were 102.0 ± 2.3%, 97.5 ± 3.9% and 97.1 ± 2.8%, respectively. 

**Table 1.** Precision and Accuracy Data for SW Voltammetric Detection of ATZ in Water Using NMCPE ^a ^

ATZ Concentration		RSD, %		RE, %
Added, ng/mL	Found, Mean ± SD	Intra-day	Inter-day	
**10**	10.2 ± 0.07	4.5	8.9	2
**30**	29.3 ± 0.14	3.7	6.6	-2.5
**60**	58.3 ± 0.17	3.2	7.8	-2.9

^a^Intra-day, n = 6; Inter-day, n = 6; Runs Per Day, Five Days

### 4.9. Real Sample Analysis

As indicated in Table 2, different amounts of ATZ were spiked into the tap and river water samples and the recovery of ATZ were obtained at optimum conditions. Recovery tests showed reliability and accuracy of the method. 

**Table 2.** Determination of ATZ Content and Recovery Tests in Tap and River Water Samples With the Proposed Method (n = 6) ^a ^

Sample	Added ATZ, ng/mL	Found ATZ^b^, ng/mL	Recovery, %
**Tap Water c**	-	ND	-
**Tap Water**	10.0	9.5 ± 0.1	95.0
**Tap Water**	50.0	50.2 ± 0.2	100.4
**Tap Water**	75.0	74.6 ± 0.3	99.5
**River Water**	-	ND	-
**River Water**	10.0	16.8 ± 0.5	96.0
**River Water**	50.0	58.0 ± 0.5	103.6
**River Water**	75.0	73.4 ± 0.4	97.8

^a^Conditions: pH = 5.5, Accumulation Time: 3 min, Conditioning Potential: -1.25 V, Conditioning Time: 5 s, Pulse Height: 25 mV; Scan Increment: 2 mV; Frequency: 60 Hz

^b^x ± s

^c^Tap Water Components: Ca^2+^ = 52, Mg^2+^ = 29, Na+ = 37; CO_3_^2-^ = 61, Cl^-^ = 24; SO_4_^2-^ = 25, NO_3_^-^ = 9 mg/mL, pH = 7.3, EC = 853

## 5. Discussion

This simple, fast and cost effective proposed methodology can be an alternative for more sophisticated methods such as immunoassay or chromatography (16 -19). Up to our best knowledge this may be the first nano-material modified carbon paste electrode used for ATZ indirect determination. In addition, this may be the most easily fabricated, cheap and sensitive modified carbon paste electrode used for ATZ detection. The determination process needs no complicated pretreatment processes such as those needed in immunosensors. LOD of the method is low and better than some of the previously reported methods. The proposed method was successfully applied for determination of ATZ in water samples. No use of toxic Hg electrode and short analysis time are other advantages of the proposed methods. Table 3 shows a comparison between analytical features of the proposed method with those of other researches. Some reported LOD values (most of these reports are chromatographic and bio-sensing methods) are better than that of the proposed method, but these methods suffer from extensive and expensive pre-treatment steps and lengthy time of analysis. The proposed method shows good and comparable recovery values with respect to other reported methods. The main drawbacks of the method are mentioned in sections 4.7 although, this developed procedure has been designed for determination of ATZ in water samples including this compound lonely, it can be considered as a new sensitive and fast method for detection of similar herbicides in water samples by chromatographic systems such as HPLC and capillary electrophoresis. In other words, although, some compounds such as simazine may do interfere but this technique can be used as HPLC or electrophresis detector. 

**Table 3.** The Comparison of the Proposed Method (SW Voltammetric Determination of ATZ by NMCPE) With Some Reported Methods of ATZ or Other Triazine Herbicides ^a ^

Method	Detection Limit, ng/mL	Analysis Time, min	Recovery, %
**GC-MS (9)**	0.0045	100	70
**GC-MS (8)**	0.12	100	87-114
**LC-FL (5)**	1.20	20	84-95
**GC-MS (10)**	0.002	30	80-120
**SPME-GC (6)**	0.001	25	72-109
**FI-HPLC (12)**	1.3	40	96-107
**HPLC-MS (15)**	1.3	30	66-88
**HPLC (16)**	3	25	87-101
**EIS (23)**	8.3	20	NR^b^
**EIS (22)**	0.003	60	NR
**V, Hg (27)**	2	10	92-116
**V, Hg (28)**	4	30	95-101
**ASV, Hg (29)**	0.024	25	95-109
**NMCPE**	2.0	10	95-104

^a^Abbreviations: GC-MS, gas chromatography–mass spectrometry; HPLC-MS, high performance liquid chromatography–mass spectrometry; LC-FL, liquid chomatography-fluorimetry; FI, flow injection; SPME, solid phase microextraction; EIS, electrochemical impedance spectroscopy; V, voltammetric; ASV, anodic stripping voltammetry

^b^Not Reported
